# Biochemical analysis of methanolic extract from *Evolvulus alsinoides*

**DOI:** 10.6026/973206300191173

**Published:** 2023-12-31

**Authors:** Karnam Nithya, Rajaram Siddaraman, PP Sheelajoice, Mani Rajarathinam, Vurimi Bhopal Chandra

**Affiliations:** 1 Department Of Pharmacology, Vinayaka Missions University, Salem (Deemed to be university), Chinna Seeragapadi, Salem - 636308, Tamilnadu, India; 2 Department of Pharmacology, VMKV Medical College, Chinna Seeragapadi, Salem - 636308, Tamil Nadu, India; 3 Department Of Physiology, VMKV Medical College, Chinna Seeragapadi, Salem - 636 308, Tamil Nadu, India; 4 Department Of Pharmacology, GMKMC Medical College, Shavapet Salem 636002, Tamil Nadu, India; 5 Department of Pharmacology, Narayana Medical College, Nellore - 524 003, AP, India

**Keywords:** Phytochemistry, *Evolvulus alsinoides*, convolvulaceae, GCMS

## Abstract

*Evolvulus alsinoides* is a medicinal plant in the Convolvulaceae family. Traditionally, it is used for different ailments in India and in several other countries. It has a variety of pharmacological qualities, including those that aid wound healing, hepato-protection, cardio-protection, anti-diabetic action, asthma, and epilepsy, memory and learning, and neuroprotection. The whole plant is utilized in Ayurvedic medicine to treat neurological disorders, including amnesia, and it is called the brain tonic by them. Therefore, the use of GC-MS in phytochemical research and chemotaxonomic investigations of medicinal plants containing physiologically active components is critical. Hence, the various secondary metabolites from the methanolic extract of *E. alsinoides* were analyzed using GC-MS technique. The methanolic extract of *E. alsinoides* yielded ten compounds. All ten compounds showed the highest number of hits. Those with the highest concentration were chosen to identify the qualitative compound. All compounds are non-toxic. Molecules with specific properties are capable of modulating a variety of proteins, including some enzymes. Thus, these molecules are potential hit-to-lead molecules in preclinical studies.

## Background:

The Convolvulaceae family member *Evolvulus alsinoides (Linn)* is also known as Vishnukranthi/Shankhpushpi. *E. alsinoides*. is typically found in grassy and open spaces. It can be found in subtropical nations, including the Philippines, India, and Africa. *Evolvulus alsinoides* are called dwarf morning glory and have light blue flowers and small leaves; fruits are like slender capsules. This plant is distributed in open grassy lands and in deep soil lands. Two varieties of *Evolvulus alsinoides* are distributed all over India. One is *Evolvulus alsinoides* (L.) L. var. angustifolius Torr. - slender dwarf morning glory and another one is *Evolvulus alsinoides* (L.) L. var. debilis (Kunth) van Ooststr. - slender dwarf morning-glory. Whole plants and leaves are the parts used for medicinal purposes [1]. In traditional systems of medicine such as Ayurveda and Unani, *Evolvulus alsinoides* Linn is utilized as a nootropic or brain-tonic. Certain ethnic races in India, Africa, and the Philippines utilize the plant to treat fever, cough, cold, venereal disorders, azoospermia, adenitis, and depression [2]. According to Ayurveda, Vishnukranthi is a key component of Medhya Rasyana (nervine tonic) herbs, which benefit synaptic plasticity and neural regeneration. Anticonvulsant, anthelmintic, sedative, memory-enhancement, antiepileptic, and anti-anxiety are among the traditional uses. The plant is also utilized for many other conditions, including epilepsy, uterine bleeding, nervous debility, and insanity. It is also used as a brain and memory tonic and for antifungal, antibacterial, antiulcer, and anti-asthmatic properties [3]. It is used as a brain tonic, vermifuge, antistress, antidepressant, anxiolytic, analgesic, neuroleptic, and anti-inflammatory herb Anti-amnesic, antistress (adaptogenic), antibacterial, and gastroprotective action has been reported in pre-clinical both in vivo and in vitro studies [4]. It is also a brain tonic that is used to improve brain function and a major ingredient in many Medhya formulations. It is of interest to restore diminishing cognitive capabilities; herbal medications are used as a traditional and alternative therapy. Secondary metabolites found in medicinal plants have a wide spectrum of therapeutic characteristics. The biochemical effects are caused by the medicinal plant's assistance in slowing essential metabolic pathways or blocking enzymes. Furthermore, herbal bioactives promise to be rewarded for their efficacy, safety, and acceptability. Medicinal plants have been identified as a viable source of lead compounds for the creation of novel drugs [6]. Gas Chromatography-Mass Spectrometry (GC-MS) aims to isolate various substances within a given sample, which is then used to retrieve the accessible compounds from the plant extract. Previous research has documented the presence of countless secondary metabolites with potent Anti-amnesic, antistress (adaptogenic), antibacterial, and gastroprotective, epilepsy, uterine bleeding, nervous debility, insanity, vermifuge, antidepressant, anxiolytic, analgesic, neuroleptic, and anti-inflammatory, antifungal, antiulcer, and antiasthmatic properties [6]. Therefore, we are interested in the isolation and phytochemical evaluation from the methanolic extract of *Evolvulus alsinoides Linn* using GC-MS analytical method, and objective was to extract the phytochemicals from the methanolic extract of *Evolvulus alsinoides* Linn using GC-MS analysis.

## Materials and Methods:

## Plant materials:

The *Evolvulus alsinoides* plant was collected in July 2022 from Sri Venkateswara University, Chittoor District, Andhra Pradesh, India, from a single herb. Dr K. Madhava Chetty, Plant taxonomist, (IAAT:337), Department of Botany, Tirupathi-Andhrapradesh, India, identified and authenticated the whole plant. Voucher number: 0669 Botanical names *Evolvulus alsinoides (L)L.,* The whole plant was cleaned with fresh running tap water followed by distilled water and dried in a shaded sunlight area after authentication, which was later finely powdered. The powdered plant was subjected to alcoholic extraction by maceration. The obtained extract was subjected to quantitative chemical analysis with GC-MS to evaluate the compounds present. We further attempted to obtain from those compounds to know their pharmacokinetic and toxicological properties and their pharmacodynamic activity.

## Preparation of plant extract:

Kahkonen et al5 described a modified approach for extract preparation. 500 mg of ground dry plant materials were weighed in a test tube, followed by 10 ml of 80% aqueous methanol. After then, the suspension was gently swirled. The tubes were sonicated for 5 minutes at 45°C before being centrifuged for 10 minutes at 1500 g at 25°C. The supernatants that resulted were collected. The extraction method was done three times, and the supernatants were mixed before being evaporated to a volume of roughly 1 ml using a rotary evaporator. After that, the concentrated extracts were lyophilized and weighed. The extracts were resuspended in saline before being utilized as a stock solution.

## Phytochemical analysis:

Phytochemical analysis was completed as described elsewhere [7](Table 2).

## Tannins:

1ml of the sample was taken, to which a few drops of 0.1 % ferric chloride was added and observed for brownish green or blue-black coloration.

## Saponins:

1 ml of sample was taken, and 2 ml of water was added. The suspension was shaken in a graduated cylinder for 15 minutes. A layer of foam indicates the presence of saponins.

## Flavonoids:

1 ml of sample was taken, that add NaOH to observe the yellow color, and concentrated hydrochloric acid was added and observed white color.

## Alkaloids:

1 ml of sample was taken, adding a few drops of Dragendorff's reagent, A prominent yellow precipitate indicates the test is positive.

## Protein:

1 ml of sample was taken, to which that few drops of Millon's reagent were added. A white precipitate indicates the presence of Protein.

## Steroids:

1 ml of sample was taken, and two drops of 10% concentrated sulphuric acid were added and observed for brown color.

## Anthraquinones:

1 ml of sample was taken, and aqueous ammonia was added and observed for color change. Pink, red, or violet color in the aqueous layer is not formed indicating absence of anthraquinones.

## Phenols:

1ml of the sample was taken; to that 3ml of 10% Lead acetate solution was added. A bulk white precipitate formed at the surface indicates the presence of phenolic compounds.

## Terpenoids:

2 ml of chloroform, followed by 3 ml of concentrated sulphuric acid, was added to 0.5 ml of the extract. The formation of red-brown color at the interface confirms the presence of terpenoids.

## Carbohydrates:

0.5ml of the sample was taken, 0.5ml of Benedict's reagent was added and mixed well, then placed in the water bath for 2mins. The colored precipitate is not formed indicatites absence of carbohydrate.

## Determination of the total flavonoid contents analysis:

The aluminium chloride colorimetric test assessed the TFC of crude bark extracts. The calibration curve was produced using a standard of quercetin (20-100 ng/mL). 1 mL crude extract was combined with 2.8 mL double distilled water and then with 0.1 mL potassium acetate solution (1 mg/mL). A UV-visible spectrophotometer was used to detect absorbance at 415 nm after adding 0.1 mL of 10% aluminium chloride to the solution and allowing it to stand for 30 minutes. The TFC was calculated using a calibration curve, and the results are given in milligrams of quercetin equivalents per gram of bark (mg/g) (dry weight) [8].

## Determination of the total phenolic content analysis:

The Folin-Ciocalteu (FC) technique was used to calculate the TPC. Gallic acid (20-500g/mL) was used as a standard to create the calibration curve. 1 mL of crude extract was diluted up to 3 mL with distilled water and carefully mixed with 1 mL of FC reagent (previously diluted 6-fold with distilled water), then 2 mL of 20 percent (w/v) sodium carbonate was added. An UV-visible spectrophotometer was used to measure absorbance at 765 nm after the mixture had been allowed to stand for 30 minutes in the dark. The TPC was calculated using a calibration curve, and the results are given in milligrams of gallic acid equivalents per gram of bark (mg/g) (dry weight) [9].

## Gas chromatography - Mass spectrometry analysis:

**Figure 1:**
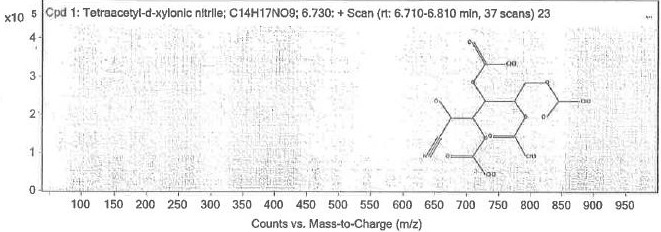
Gas chromatography-mass spectrum(GC-MS) analysis chromatogram of isolated compounds from *E.alsinoides* showing 5 distinctive peaks time and relative abundance plotted along x axis and y axis respectively.

Analysis of *Evolvulus alsinoides* extract was carried out using GC-MS equipment Figure 1. The GC-MS system used a TR 5MS capillary standard non-polar column with a diameter of 30 Mts, an ID of 0.25 mm, and a film thickness of 0.25 m. The flow rate of the mobile phase was set to 1.0 mL/min from the start. In the gas chromatography section, the temperature was raised from 40°C to 250°C at a rate of 5°C/min, with an injection volume of 1 microliter. The Wiley Spectral library search tool was used to analyze the outcomes of the samples immersed in chloroform over a mass spectrum of 50650 m/z12 [10].

## Results:

A total of 10 compounds were identified in the GC-MS analysis, out of which five compounds show significance (2 compounds having two peaks) and out of 10 compounds, five compounds had more hits; the obtained chromatogram was presented. The compounds with a greater number of hits were evaluated for pharmacodynamic properties. Table 1 depicts the availability of various compounds in the methanolic extract of *E.Alsinoides*, which may be important for the pharmacodynamics and pharmacokinetic potency and their general physicochemical properties. A total of 10 compounds are seen in the chromatogram. Still, only five compounds are predominantly observed as productive based on the area and peak obtained in the chromatogram. They may be responsible for the pharmacological actions of the methanolic extract of *E.alsinoides.*

**Figure 2:**
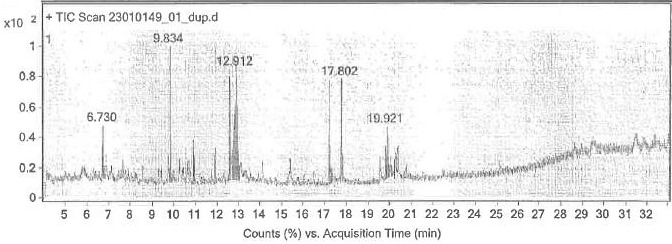
Structure profiling of the compounds identified as Tetraacetyl-d-xylonic nitratrile from the *E. Alsinoides* using GC-MS

**Figure 3:**
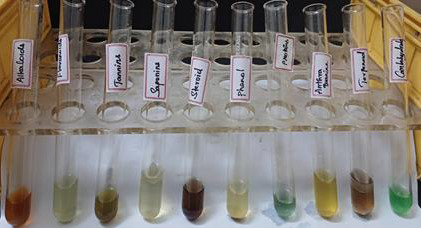
Phytochemical analysis

The peak obtained at 6.73 retention time, as per mass spectra details, represents the chemical tetra acetyl-d-xylonic nitrate - compound Figure 2. The peak obtained at 9.834 retention time, as per mass spectra details, represents the chemical Bicyclo [5.2.0] nonane 2methylene 4,8,8trimethyl-4vinyl- compound. The peak obtained at 10.909 retention time, as per masss spectra details, represents the chemical Benzoic acid, 4 -ethoxy-ethyl ester - compound. The peak obtained at 11.917 retention time, as per mass spectra details, represents the chemical cls-5,8,11,14,17, Eicosapentaenoic acid compound. The peak obtained at 12.591 retention time, as per ms details, represents the chemical triethyl citrate compound. The peak obtained at 12.912 retention time, as per mass spectra details, represents the chemical 3-o-methyl-d-glucose compound. The peak obtained at 17.244 retention time, as per mass spectra details, represents the chemical n-hexadecanoic acid compound. The peak obtained at17.802 retention time per mass spectra details represents the chemical hexa decanoic acid, ethyl ester compound. The peak obtained at 19.831 retention time, as per mass spectra details, represents the chemical 8,11,14, Eicasa trienoic acid -(Z, Z, Z)- compound. The peak obtained at 19.921 retention time, as per mass spectra details, represents the chemical 8,11,14, Eicasa trienoic acid -(Z, Z, Z)- compound (Table 3). Further, phytochemical screening was done to find the presence of flavonoids, alkaloids, tannins and steroids and glycosides (Table 2 and Figure 3).

## Discussion:

Secondary metabolites important and rich in many medicinal plants include phenols, saponins, tannins, alkaloids, flavonoids, and glycosides. GC-MS can characterize these metabolites; they are considered the primary source of biological and pharmacological activities.

There are many Pharmacological uses and medicinal properties for *E. alsinoides*E, such as Anticonvulsant properties, The plant's anticonvulsant and hypnotic properties were investigated using crude methanolic extract at dosages of 50, 100, 200, and 400 mg/kg in mouse models for maximal electroshock seizures and pentylenetetrazole-induced seizures, as well as a diazepam-induced sleep paradigm. The combination of 400 mg/kg of the extract plus 30 mg/kg of diazepam provided the strongest anticonvulsant effects. These results support using the plant's methanolic extract in managing epilepsy. Anti-inflammatory activity: In acute toxicological investigations, the chloroform and ethyl acetate extracts at 200 and 400 mg/kg body weight demonstrated graded dose-response. The ethanolic extract significantly inhibited the anti-inflammatory action [11].

Antimicrobial property, when acetone extract was used, *EE. alsinoides (L.) L*E showed antimicrobial activity against *Acinetobacter baumannii*, *Aspergillus niger*, *Cryptococcus neoformans*, and *Candida albicans*, as well as mild activity against *Bacillus subtilis*, *Klebsiella pneumonia*, Pseudomonas aeruginosa, and Staphylococcus aureus. The extracts' inhibitory effects were equivalent to those of the conventional antibiotic. Overall, they are more effective as antifungal agents than antibacterial ones. Anxiolytic property, Anxiolysis was seen in an elevated plus maze test using ethyl acetate fractions of the plant extract. A larger dose of 200 mg/kg resulted in a significant decrease in rotarod performance. The aqueous fraction showed no such response. The ethanolic extracts showed neuromuscular coordination and strong antioxidant potential [12].

Cardio-protective Effects were seen with E. alsinoides when the methanolic extract was used to study the mitigation of acute myocardial infarction in an isoproterenol [ISP]-treated rat model while maintaining cardiac function and activities of natural antioxidant enzymes. Male albino rats were used for the biochemical examination of the serum plasma and the enzyme analysis of the heart tissue. The findings show cytoprotection with plant extract at 100 and 200 mg/kg/p.o doses induce myocardial adaptation by augmenting endogenous antioxidants and guards against oxidative stress associated with ISP-induced myocardial injury. The outcomes support any therapeutic benefit in the management of ischemic heart conditions [13].

Effect on the pancreas in streptozotocin-induced diabetic rats showed considerable antioxidant activity with increased insulin levels and suppressed lipid peroxidation after receiving plant extract orally for 45 days. Due to the presence of secondary metabolites with therapeutic potential in the ethanolic extract of the plant, administration of the plant extract to the research animals enhances antioxidant activity and changes the structure of the pancreas22. These results imply that using plant extract significantly decreased the oxidative stress brought on by streptozotocin and may have raised insulin levels. Administration of the plant extract can prevent the onset of diabetes mellitus [14]. Nootropic activity is predominantly seen with Alcoholic extracts of *E. alsinoides (L.) L* has been shown to have better nootropic activity in terms of time spent in the enclosed arm and mean avoidance reaction in the plus maze and jumping box models, respectively. According to the research, plant extracts can be cognitive enhancers to boost cerebral function [15].

Anti-leukemic activity has been noticed while observing the cytotoxic effects of the ether and methanol extract on the cell lines HL60, K562 and U937, revealing that Kaempferol-3, 7-di-O-rhamnoside has the strongest anti-leukemic properties. The report of its cytotoxic effects suggests using the plant extract to treat tumors [16]. The antioxidant capacity of plant material is determined by its functional groups. Using a single approach to assess and correlate the antioxidant activity of substances is ineffective. The 2,2-diphenyl-1-picrylhydrazyl assay (DPPH) and the Ferric Reducing of Antioxidant Power Assay were significant radical scavenging assays (FRAP). Soluble fractions were produced at concentrations ranging from 0.025 to 0.5 mg/mL for the testing. The IC50 values for each extract were calculated by graphing the inhibition percentage versus the sample concentration [17].

*Evolvulus alsinoides* antimicrobial properties are observed with methanolic extracts. *Candida albicans*, *Aspergillus niger*, *Staphyloccus aureus*, and *E. alsinoides (L.) L.* methanol extract is the organism utilized to test the antibacterial activity. *Listeria monocytogenes*, *Yersinia enterocolitica*, *Vibrio cholera*, *Bacillus megaterium* *Klebsiella pneumonia*, *Salmonella typhi*, and *Bacillus subtilis* were also tested. The methanol extract of *E. alsinoides (L.) L.* yielded the inhibitory values for each microorganism at three distinct doses. There are three distinct concentrations: 50, 100, and 150 g/ml [18].

Many therapeutic uses have been discovered for *Evolvulus alsinoides*. It primarily treats diabetes, ascites, gastric distress, flatulence, anxiety neurosis, stress condition, intestinal colic, piles, backache, and migraine. It is also used to treat common colds, coughs, asthma, and skin eruptions. Because it has a soothing impact on the brain, it treats sleeplessness, irritability, and epilepsy. It is a natural sedative that promotes restful and rejuvenating sleep. This remedy is recommended when the mind becomes overworked, agitated, and restless. It has a mild laxative effect and aids digestion. It benefits both the male and female reproductive organs [19]. It's a revitalizing plant with anti-aging benefits [20-21,22, 23,24,25, 26]. It may also aid in preventing alterations in neuron cell bodies in specific brain locations. It is also beneficial in cases of fever, asthma, bronchitis, and hypertension. It is a powerful medicine for digestive disorders, particularly diarrhea and dysentery. It has been demonstrated to be beneficial in lowering several types of stress, including psychological, chemical, and traumatic stress [22].

## Conclusion:

Various secondary metabolites from the whole plant of methanolic extract of *E. alsinoides* were analysed using GC-MS. The methanolic extract of E. alsinoides yielded ten compounds. All ten compounds showed the highest number of hits. Those with the highest concentration were chosen to identify the qualitative compound. All compounds are non-toxic. Molecules with specific properties can modulate various proteins, including some enzymes. Thus, these molecules are potential "hit" to "lead" molecules in preclinical studies.

## Ethical Considerations:

The study was performed in accordance with the universal ethical principles stated in the Declaration of Helsinki on human research.

## Code of ethics:

The study proposal was reviewed and approved by the ethical committee of ACS Medical college and hospital, Dr. MGR educational and research institute (Ethical approval code: VI/IAEC/DrMGR/2053/PO/ReBi/S/19/CPCSEA/28.01.2023/03).

## Authors' contributions:

Study design: KN. Data gathering: KN. Data Analysis: VBC, SR. Drafting the manuscript: PS. Revising the Manuscript: RM. Final Approval: SR, PS, RM.

## Figures and Tables

**Table 1:** Mass spectrospcopy identified compounds.

**Retention Time**	**Compound Name**
6.73	Tetraacetyl-d-xylonic nitrate
9.834	Bicyclo[5.2.0]nonane 2-methylene 4,8,8-trimethyl-4-vinyl
10.909	Benzoic acid, 4-ethoxy-ethyl ester
11.917	Omega-3 fatty acid (EPA)
12.591	Triethyl citrate
12.912	3-O-methyl-d-glucose
17.244	Palmitic acid (C16:0)
17.802	Palmitic acid ethyl ester
19.831 & 19.921	Eicosatrienoic acid (Z,Z,Z)

**Table 2:** The Phytochemical studies of the sample

**Test**	**Result**
TANNINS	Positive
SAPONINS	Positive
FLAVONOIDS	Positive
ALKALOIDS	Positive
PROTEINS	Positive
STEROIDS	Positive
ANTHRAQUINONES	Negative
PHENOLS	Positive
TERPENOIDS	Positive
CARBOHYDRATES	Negative

**Table 3:** Total Phenolic Content (TPC) and Total Flavonoid Content (TFC) of methanolic extract of Evolvulus alsinoides

**Extracts**	**TPC (mg of GAE/g)**	**TFC (mg of QE/g)**
Methanolic extract of Evolvulus alsinoides	35.7±0.78	38.8±1.42
